# Love, labor and loss on the frontlines: India’s community health workers straddle life and the COVID-19 pandemic

**DOI:** 10.7189/jogh.11.03107

**Published:** 2021-12-25

**Authors:** Baldeep K Dhaliwal, Shalini Singh, Lexy Sullivan, Preetika Banerjee, Rajeev Seth, Paramita Sengupta, Ipsita Bhattacharjee, Kayur Mehta, K Srinath Reddy, Anita Shet

**Affiliations:** 1 Johns Hopkins Maternal and Child Health Center India, Kolkata, West Bengal, India; 2 International Vaccine Access Center, Johns Hopkins Bloomberg School of Public Health, Baltimore, Maryland, USA; 3 Bal Umang Drishya Sanstha, New Delhi, India; 4 Department of Community Medicine and Family Medicine, All India Institute of Medical Sciences, Kalyani, West Bengal, India; 5 Child in Need Institute, Daulatpur, West Bengal, India; 6 Public Health Foundation of India, New Delhi, India

The COVID-19 pandemic has highlighted the indispensability of frontline health workers while simultaneously revealing their immense challenges. The second surge of COVID-19 brought catastrophic consequences in rural India, home to 800 million people, and exacted an immeasurable toll on over two million community health workers (CHWs) [1-3]. Misleadingly called ‘volunteers’, CHWs, who are usually women from communities they serve, support health promotion and prevention which form the backbone of rural primary health care [4-6]. Limited research has been conducted on understanding how the pandemic impacted the professional and personal experiences of CHWs in India during the pandemic.

## UNDERSTANDING THE IMPACT OF THE COVID-19 PANDEMIC ON COMMUNITY HEALTH WORKERS

To understand the impact of the COVID-19 pandemic from community health workers’ perspectives, we interviewed three cadres of female CHWs across India: Accredited Social Health Activists (ASHAs), Auxiliary Nurse Midwives (ANMs), and Anganwadi Workers (AWWs). These types of CHWs were selected as they have close relationships with members of the community and have worked in community settings throughout the pandemic. Through partners in India, we used purposive sampling to identify 45 CHWs across seven states (Haryana, Jharkhand, Karnataka, Madhya Pradesh, Nagaland, Uttar Pradesh, and West Bengal). Our sampling strategy allowed us to use past experiences and knowledge of communities to identify CHWs who were well-positioned to provide in-depth information about their experiences during the COVID-19 pandemic. These CHWs were then interviewed using a standard interview guide, which was developed to be aligned with the research questions and aims. Interviews were conducted by data collectors who had experience in quantitative and qualitative data collection. Data collectors were provided with additional training on specific qualitative techniques such as probing and open-ended questions. Domains covered in the interview guide included: (1) personal experiences, (2) work experiences, and (3) long-term support and changes needed; probes were used to ask follow-up questions and identify additional details. Data collectors documented responses to open-ended questions. Two independent readers conducted rapid qualitative evaluation and thematic analysis on the interview responses to develop an understanding of the situation from the perspective of the CHWs. Interview responses were summarized into categories using a standard template across settings. These summary tables were consolidated to identify barriers, strengths, and areas for improvement to strengthen our understanding of CHW experiences across multiple settings.

**Figure:**
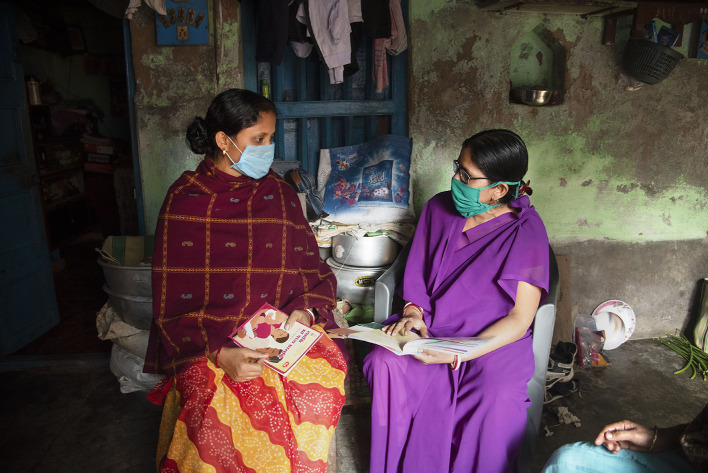
Photo: From the collection of the Maternal and Child Health Center, credit: Paramita Chatterjee.

## CHALLENGES IDENTIFIED AMONG COMMUNITY HEALTH WORKERS

### Increased workload

CHWs across all cadres reported a substantially increased workload during the pandemic. They described working with negligible breaks and no fixed hours for the past year; some ASHAs explained that their usual 3-4 hours per day increased 3-fold during the pandemic.

“The change in responsibility is also there, like if something [COVID-related] happens within the village, the visits start [to] happen. Being [a CHW] I have to be present there for each and every visit of senior officials. This is more extensive than [my] previous workload.”

CHWs’ tasks shifted towards COVID-19, with duties that included promoting COVID-19 preventive behaviors, contact tracing, mobilizing testing, ensuring home quarantines, delivering medicines, and supporting COVID-19 vaccination drives. Balancing their pandemic-related workload and other essential services proved challenging; some indicated that as COVID-19 vaccinations took center-stage, routine immunization activities decreased, potentially leaving pregnant women and children vulnerable to vaccine-preventable illnesses.

“Most of my time is now dedicated to COVID-related home visits [not routine care], health promotion activities, community surveillance and contact tracing, monitoring home quarantine, facilitating access to diagnostics.”

Travel restrictions and public fear also prevented the provision of services such as home-based newborn and antenatal care.

### Reduced financial support

Paradoxically, despite additional responsibilities, CHWs’ earnings declined. In India, many CHWs receive service-based incentives for specific duties such as facilitating routine immunization and institutional deliveries, activities that declined significantly during the pandemic [7]. CHWs’ new COVID-related work was tied to low financial compensation with state-specific variations in the disbursement of incentives.

“Mobilizing pregnant women for institutional delivery is [a] major part of [our] work, but they are not opting to use government health services due to COVID-[related] fear[s]. So, Janani Suraksha Yojana incentives have greatly reduced.”

Interviewees also explained that their family members had lost jobs, increasing the families’ dependence on the CHWs’ inadequate salaries, and deepening economic hardship.

“My husband has lost his employment due to which financial stress has increased. We were enjoying our work before. Now there is a constant fear.”

### Impacts on mental health

Like other health care workers navigating the pandemic, CHWs shared their increased feelings of frustration, loneliness, and loss, sentiments magnified by their lack of empowerment in the community. Many self-isolated to protect their families but still lost loved ones to COVID-19. Several expressed anguish about facing stigma related to their exposure to COVID-19 and subsequent shunning by community members.

“Initially… people were not ready to talk with us because maybe I am a [COVID]-affected person, [maybe] I can infect them. We were working for the community despite thinking about our own life, but people don’t want us. Let me be honest with you, that was very painful initially.”

### Overcoming challenges

Despite these struggles, a sense of optimism and pride for their roles shone through. The CHWs’ narratives reflected their deep love for their communities that propelled their commitment to providing care. They often exceeded expectations, as illustrated by one ASHA who brought a woman with cardiac disease into her home until they could facilitate hospital admission during the nationwide bed shortage. Additionally, CHWs emerged as champions in the COVID-19 vaccination drive due to their own high uptake of COVID-19 vaccines.

Braving hardship, CHWs demonstrated ingenuity and adaptability in assisting communities. In an innovative turn of responsibilities, AWWs who were unable to cook food for children at Anganwadi centers, conducted home delivery of dry rations for children and pregnant women. CHWs formed new partnerships with local non-health organizations, becoming foot soldiers of the nation by protecting and supporting citizens.

### Unmet needs

In their efforts to provide care to their communities, CHWs faced several systemic barriers. They expressed the need for greater support from supervisors to navigate increased workloads and manage COVID-19 stigma. Regular guidance from experts, consistent health care in the event of illness, and endorsement from community leaders would increase their effectiveness.

“The influential community members should support us in doing our work. Then the community will support us.”

CHWs spoke in unison about needing recognition of their roles, empowerment within their communities, and inclusion in local health-related decision-making. They emphasized the benefit of mental health services to address increased anxiety and stress. Finally, CHWs reflected on the urgent unmet need of sustainable financial support to improve their quality of life.

“For an ASHA worker, work is too much but salary is less. I [had to] do stitching work in order to contribute financially… We [ASHAs] suffer financially most [during COVID].”

## WAYS FORWARD

### Key issues

We identified several key issues from our interviews with CHWs across India. CHWs faced increased workloads, decreased compensation, and stated that their work had shifted to focus on COVID-related work, as opposed to routine care. CHWs also shared that their needs included improved mental health services, financial payment that was not tied to incentives, and consistent access to PPE. To date, CHW experiences through the context of the COVID-19 pandemic have not been well-explored. Uniquely situated between the community and the health care system, CHWs are indispensable in maintaining the health of the nation, and in confronting current and future pandemic [8]. As such, it is critical to understand their perceptions of health care delivery.

### Adapting learnings from CHW experiences during prior outbreaks

The role of the CHWs was critical in providing routine care and ensuring trust during the Ebola outbreak in Guinea, Liberia, and Sierra Leone [9]. However, due to their late engagement by the government, their ability to respond to the Ebola outbreak was significantly hindered. After the government engaged CHWs, relationships between CHWs and community members proved to be incredibly resilient. During the most critical period of the Ebola outbreak, CHWs were found to be more effective at carrying out Ebola-related activities than other individuals who were not from the community. This demonstrates the need for local CHWs to be placed at the forefront of emergency and pandemic preparedness and response plans, and to ensure that they are adequately provided with the support needed to strengthen the delivery of health services.

### Leveraging CHW expertise to facilitate long-term change

Acknowledging the power of CHWs to prevent disease in vulnerable communities, as well as providing them with the resources needed to empower them, may improve health outcomes for the most marginalized individuals across India. This is particularly important to consider given the dual burden of disease in India where non-communicable diseases (NCDs) are rising in conjunction with constant threats from emerging and re-emerging infectious diseases. Although CHWs have contributed greatly to maternal, neonatal, and child health, their roles have gradually expanded in prevention, screening, and care coordination for infectious diseases as well as NCDs [10,11]. By working closely with CHWs in India, as trusted members of the community, health officials may be able to address emerging and threatening health conditions more effectively.

Although the vignettes presented here are limited by small numbers and uncertain generalizability, governments would do well to recognize CHW’s critical role, as demonstrated by the WHO’s declaration of 2021 as the International Year of Health and Care Workers [12]. The National Human Rights Commission of India has also circulated recommendations on health rights of health workers to federal and state governments [13].

## CONCLUSIONS

Our study highlights the difficulties faced by CHWs in India during the ongoing COVID-19 pandemic. Providing mental health and financial support, in addition to basic needs such as PPE, will further enable CHWs to continue to serve their communities. Attention to these needs of CHWs is crucial when state and federal governments take concrete social and political commitments to improve implementation of health services at regional levels . During this unprecedented public health crisis, CHWs have amply demonstrated that they may be our best hope for a more resilient health system and a healthier society.
